# Efficient Production of Pyruvate Using Metabolically Engineered *Lactococcus lactis*

**DOI:** 10.3389/fbioe.2020.611701

**Published:** 2021-01-06

**Authors:** Fan Suo, Jianming Liu, Jun Chen, Xuanji Li, Christian Solem, Peter R. Jensen

**Affiliations:** ^1^Division of Production and Microbiology, National Food Institute, Technical University of Denmark, Lyngby, Denmark; ^2^Section of Microbiology, Department of Biology, University of Copenhagen, Copenhagen, Denmark

**Keywords:** high-yield pyruvate production, *Lactococcus lactis*, metabolic engineering, dairy side-stream, fermentation

## Abstract

Microbial production of commodity chemicals has gained increasing attention and most of the focus has been on reducing the production cost. Selecting a suitable microorganism, which can grow rapidly on cheap feedstocks, is of key importance when developing an economically feasible bioprocess. We chose *Lactococcus lactis*, a well-characterized lactic acid bacterium, as our microbial host to produce pyruvate, which is a commodity chemical with various important applications. Here we report the engineering of *Lactococcus lactis* into becoming an efficient microbial platform for producing pyruvate. The strain obtained, FS1076 (MG1363 Δ^3^*ldh* Δ*pta* Δ*adhE* Δ*als*), was able to produce pyruvate as the sole product. Since all the competitive pathways had been knocked out, we achieved growth-coupled production of pyruvate with high yield. More than 80 percent of the carbon flux was directed toward pyruvate, and a final titer of 54.6 g/L was obtained using a fed-batch fermentation setup. By introducing lactose catabolism into FS1076, we obtained the strain FS1080, which was able to generate pyruvate from lactose. We then demonstrated the potential of FS1080 for valorizing lactose contained in dairy side-streams, by achieving a high titer (40.1 g/L) and high yield (78.6%) of pyruvate using residual whey permeate (RWP) as substrate. The results obtained, show that the *L. lactis* platform is well-suited for transforming lactose in dairy waste into food-grade pyruvate, and the yields obtained are the highest reported in the literature. These results demonstrate that it is possible to achieve sustainable bioconversion of waste products from the dairy industry (RWP) to valuable products.

## Introduction

Pyruvic acid (pyruvate), the simplest of the α-keto acids, serves a key role in living organisms. Pyruvate has many important applications, e.g., as a food supplement and as a precursor for chemicals and pharmaceuticals, and the demand for this compound has greatly increased over the past few decades (Park et al., 1998; Rosche et al., 2001; Zelić et al., 2003; Reiße et al., 2016). Pyruvate can be made by classical chemical synthesis or by microbial fermentation, where the latter approach is preferred, especially for sensitive applications such as use in food (Li et al., 2001; Stottmeister et al., 2005; Maleki and Eiteman, 2017). Chemically synthesized pyruvate is expensive, and Li et al. estimated the total cost of production from tartaric acid by dehydration and decarboxylation to be US 8,000–9,000/ton (Li et al., 2001). The fermentative approach for producing pyruvate was developed much later, probably due to the difficulties associated with getting microorganisms to secrete a central metabolite (Li et al., 2001). In the late 1980s, Toray Industries in Japan submitted several patent applications for fermentative production of pyruvate using the multi-vitamin auxotrophic yeast *Candida glabrata* (previously named *Torulopsus glabrata*) (Miyata et al., 1989a,b,c, 1990), and *C. glabrata* continues to be one of the best pyruvate producers available. The *C. glabrata* strains used are auxotrophic for thiamine (vitamin B1), nicotinic acid, biotin and pyridoxine (vitamin B6), and when grown with limiting amount of these vitamins start to secrete large amounts of pyruvate due to low activities of pyruvate dehydrogenase, pyruvate decarboxylase (PDC), pyruvate carboxylase (PC), and transaminase (Li et al., 2016). *C. glabrata* is a good pyruvate producer as it possesses a high tolerance to low pH, which lowers product recovery costs, and since it can grow on relatively simple and cheap media (Li et al., 2016), albeit with a low growth rate. Using a pH-controlled fermentation setup and an osmotolerant *C. glabrata* mutant, Liu et al. achieved a high titer of 94.3 g/L, however, the fermentation lasted 82 h and the yield was fairly low (0.635 g pyruvate/g glucose), resulting in a low productivity of 1.15 g L^−1^ h^−1^, despite using a large inoculum of 10% (Liu et al., 2007). Miyata and Yonehara achieved a somewhat lower titer of 67.8 g/L after an almost 70 h fermentation using a medium containing soy bean hydrolysate, and the yield was less than 50% (Miyata and Yonehara, 1996). There is thus great opportunity for improving the fermentation process, perhaps by using other organisms. There have been several efforts aiming to achieve pyruvate production in prokaryotes. *E. coli* is commonly used as a production host for various compounds, mainly due to its well-characterized metabolism and the plethora of genetic tools available for its manipulation. Yokota et al., using a lipoic acid auxotroph of *E. coli*, managed to produce 25.5 g/L with a 51% yield in 32–40 h, in a pH-controlled fermentation using a mineral medium, however, a 4% inoculum grown in rich medium was needed (Yokota et al., 1994). *E. coli*, despite of its ease of manipulation, is not always a suitable production host, especially for organic acids for which its tolerance is low.

Lactic acid bacteria (LAB) are immensely important within food manufacturing, e.g., the cheese bacterium *Lactococcus lactis* is annually inoculated into more than 100 million tons of milk (Teuber, 1995). Recently, LAB have been widely engineered into producing various valuable biochemicals (Ghaffar et al., 2014; Liu et al., 2016a,b,c,d). Because these bacteria rely on a purely fermentative metabolism, they usually have a high glycolytic capacity. They are also able to metabolize a broad range of carbohydrates, and are becoming increasingly easy to manipulate genetically (Kleerebezem et al., 2000; Liu et al., 2017). They naturally thrive in carbohydrate-rich environments, and usually produce lactic acid as the main fermentation product. In this study, we explored the potential of *Lactococcus lactis*, the model organism of LAB, as a pyruvate producer. We rerouted its metabolism from lactate to pyruvate, characterized its performance in various media and optimized the fermentation. We successfully managed to produce pyruvate from glucose, and also from lactose contained in dairy waste. We found that *L. lactis* has a high potential for pyruvate production, and the fermentation process developed should have industrial relevance.

## Materials and Methods

### Strains and Plasmids

The bacterial strains and plasmids used in this study have been listed in Table 1. *L. lactis* subsp. *cremoris* MG1363 (Gasson, 1983) or derivatives were used as described in this article. The plasmid pCS1966als, a derivative of plasmid pCS1966 (Solem et al., 2008), was used to delete *als* in *L. lactis*. The plasmid pCS4564 with a thermosensitive replicon carrying *E. coli ldh* (Liu et al., 2016a), was used to increase transformation efficiency of *L. lactis* strains deficient in lactate dehydrogenase. The plasmid pLP712, which carries the lactose utilization gene cluster, was obtained from the dairy isolate NCDO712 (Wegmann et al., 2012). pLC17 (Dorau et al., 2019) contains the *als* gene from *Lactococcus lactis* MG1363, and was used to re-introduce the *als* gene into FS1072.

**Table 1:** Strains and plasmids.

**Designation**	**Genotype or description**	**References**
***L. lactis*** **strains**		
CS4363	MG1363 *Δ^3^ldh Δpta ΔadhE*	Solem et al., 2013
FS1068	MG1363 *Δ^3^ldh Δpta ΔadhE* pCS4564	This work
FS1069	MG1363 *Δ^3^ldh Δpta ΔadhE Δals* pCS4564	This work
FS1072	MG1363 *Δ^3^ldh Δpta ΔadhE Δals*	This work
FS1073	MG1363 *Δ^3^ldh Δpta ΔadhE Δals* pLC17	This work
FS1076	Adaptive mutants derived from FS1072	This work
FS1080	FS1076 with pLP712	This work
**Plasmids**		
pCS4564	pG^+^host8::SP-*ldhA* (*E. coli*)	Liu et al., 2016a
pCS1966	The selection/counter selection vector	Solem et al., 2008
pCS1966als	Plasmid used for deleting *als*	This work
pLP712	Plasmid from *L. lactis* NCDO712 encoding lactose utilization gene custer	Wegmann et al., 2012
pLC17	pLC0 inserted with the *als* gene from *Lactococcus lactis* MG1363	Dorau et al., 2019

### Growth Experiments

*L. lactis* was grown at 30°C, either in rich M17 broth (Oxoid TM) and defined SAL medium (Jensen and Hammer, 1993) supplemented with glucose (Terzaghi and Sandine, 1975) and various concentrations of hemin. The compositions of both media can be found in Supplementary Table 5. When needed, antibiotics were added to the cultures in the following concentrations: 200 mg/mL erythromycin for *E. coli* and 5 mg/mL erythromycin for *L. lactis*; 8 mg/mL tetracycline for *E. coli* and 5 mg/mL tetracycline for *L. lactis*.

### Analytical Methodology

To monitor cell growth, the optical density at 600 nm (OD600) was measured. Glucose, lactate, and pyruvate were quantified using an Ultimate 3000 high-pressure liquid chromatography system (Dionex, Sunnyvale, USA) equipped with a Shodex RI-101 detector (Showa Denko K.K., Tokyo, Japan) and a diode array detector (DAD-3000, Dionex, Sunnyvale, USA), where glucose and lactate were detected using the former and pyruvate using the latter detector. The column used was an Aminex HPX-87H. The oven temperature was set to 60°C, and the flow rate of the mobile phase, which consisted of 5 mM H_2_SO_4_, was set to 0.5 mL/min.

### DNA Techniques

All manipulations were carried out according to Sambrook and Russell (2001). *E. coli* cells were transformed using electroporation. *L. lactis* cells grown in GM17 medium containing 1% glycine were made electrocompetent and transformed by electroporation according to Holo and Nes (1989). Gene deletions were achieved using the method developed by Solem et al. (2008). The plasmid pCS1966als was employed to delete *als* gene in *L. lactis*. When deleting *als* gene, ~800 bp regions upstream and downstream of the deleted *als* gene were amplified by PCR and inserted into the plasmid pCS1966. The resulting plasmid pCS1966als was then transformed to *L. lactis*, where integration resulted in erythromycin resistance. Subsequently, counterselection was carried out in the presence of 5-fluoroorotic (5FO), where excision and loss of the plasmid resulted in 5FO resistance (Solem et al., 2008). The upstream region was amplified by using the primers 5′- CTAGTCTAGATTATATAAAATCGCTCTTCTTTATG-3′ and 5′- AAAACTGCAGTTTTTATTTTACCTCTATTTGTTC−3′. The primers used to amplify the downstream region were 5′- AAAACTGCAGTAAAAAACAAGCAAATTGTGAAAT−3′ and 5′- CGGGGTACCTATTTCTTGATCTAGCTGATTAAA -3′.

### Strain Construction

Plasmid pCS1966als for deleting *als* was constructed as described above. To assist further manipulations, plasmid pCS4564 with a thermosensitive replicon carrying *E. coli ldh* (Liu et al., 2016a), was transformed into CS4363 inducing FS1068. The *als* gene deletion strain was named FS1069 (MG1363 Δ^3^*ldh* Δ*pta* Δ*adhE* Δ*als* pCS4564). The plasmid pCS4564 was lost by incubation at 36°C in the presence of 5 μg/mL hemin and the resultant strain was FS1072 (MG1363 Δ^3^*ldh* Δ*pta* Δ*adhE* Δ*als*). We further obtained the mutant strain FS1076 through adaptive evolution of FS1072. Moreover, plasmid pLP712 was introduced into strain FS1076 to generate FS1080, which enables the use of residual whey permeate (RWP) as feedstock.

### Adaptive Evolution

The strain FS1072 was re-streaked, and a single colony was used as a starting point for the adaptive evolution experiment. The adaption was carried out in a 15 mL centrifuge tube filled with 5 mL SAL medium, supplemented with 1% glucose. Every 24 h, 500 μL of the culture was transferred into a new tube containing fresh medium. Every 7 days, samples were taken from the growing culture, and streaked on an SAL plate, and the largest colony was inoculated into a new tube, whereafter the evolution experiment continued. The entire experiment lasted 21 days.

### Pre-culture

For the pre-culture, the strains were grown in 250 mL Erlenmeyer flasks with 25 mL M17 medium supplemented with different concentrations of hemin (0–1 μg/mL) and 1% (w/v) glucose. The bacterial cultures were grown at 200 rpm, 30°C. Alternatively, for strain FS1080, pre-culturing was carried out in diluted residual whey permeate (RWP) containing 1% lactose, and supplemented with yeast extract (0.5–2% w/v). The cultures were grown aerobically with 200 rpm shaking in 250 mL Erlenmeyer flasks at 30°C.

### Pyruvate Fermentation

All the fermentation experiments were performed in a 500 mL maximum working volume bioreactor (Sartorius Biostat Q) equipped with standard control units for stirring speed, temperature, pH, aeration, etc. The pH was adjusted to 7.0. All the fermentation experiments were performed at 30°C with a constant aeration rate of 0.2 vvm and a constant stirring speed of 150 rpm. The pH and dissolved oxygen were monitored during the fermentation, and samples were withdrawn regularly to determine glucose, cell density (OD600) and pyruvate concentration.

### RNA Analysis

All the RNAseq data analysis in the study were done after data trimming. These included mapping, gene reads counts, normalization, statistical analysis, and gene annotation, and this was done using R (R Development Core Team, 2011). After trimming the reads and checking the quality, we mapped the clean reads to the reference Genome (GenBank accession number: NC_009004) using the function “align” in the Bioconductor package “Rsubread” (Liao et al., 2019). The R function “featureCounts” in the package “Rsubread” was used to summarize the reads and give the number of reads mapped per gene. Before proceeding with the differential expression analysis, we filtered out genes that were expressed to a very low level, meaning less than 1 count-per-million (CPM). Calculating the CPM values were done using the R function “cpm” in the package “edgeR” (Robinson et al., 2009). The R function “voom” in the package “limma” (Ritchie et al., 2015) was used to normalize the read counts as log2CPM and apply a linear model to the normalized data for computing moderated t-statistics of differential expression. The R function “eBayes” in the package “limma” (Ritchie et al., 2015) computed moderated t-statistics of differential expression (DE). The R “topTable” function in the package “limma” (Ritchie et al., 2015) summarized the DE genes in a table format. The annotation of gene IDs was done manually using the reference genome GFF (General Feature Format) file in R, which consists of the gene annotations. The RNA-Seq data have been deposited at the Sequence Read Archive (SRA) under the accession number PRJNA 669338. https://www.ncbi.nlm.nih.gov/bioproject/PRJNA669338/.

## Results

### Construction, Characterization, and Adaptive Evolution of the Pyruvate-Producing *Lactococcus lactis* Strain

**Figure 1:**
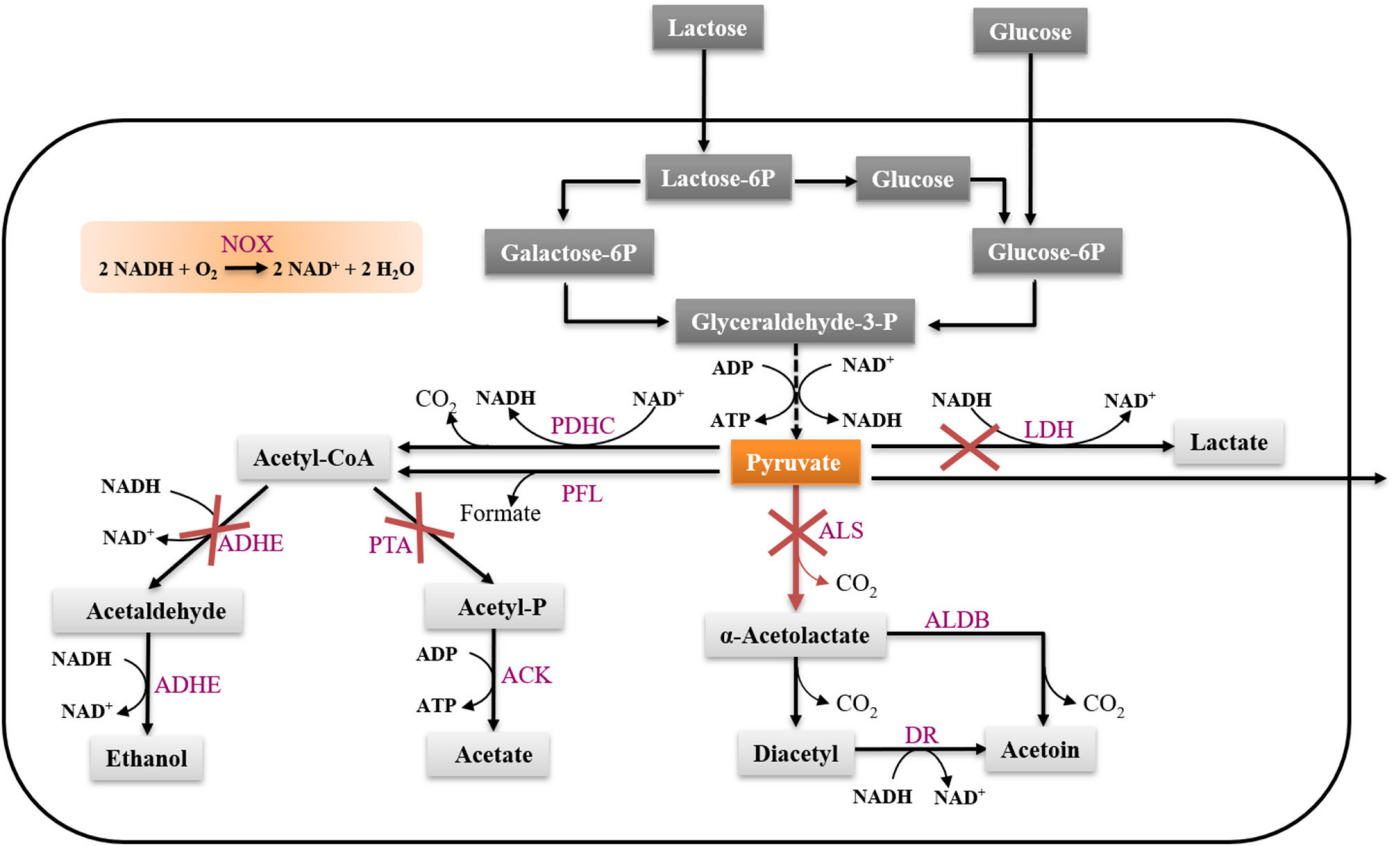
Metabolism of lactose and glucose in *L. lactis*. LDH, lactate dehydrogenase; ALS, α-acetolactate synthase; ALD, α-acetolactate decarboxylase; PDHc, pyruvate dehydrogenase complex; PFL, pyruvate formate lyase; PTA, phosphotransacetylase; ADHE, alcohol dehydrogenase; ACK, acetate kinase; Nox, NADH oxidase; DR, diacetyl reductase.

Pyruvate is a central metabolite that some microorganisms produce in small amounts, usually due to leakage across the cell membrane (Maleki and Eiteman, 2017). Efficient microbial production of pyruvate usually requires a limitation in the catabolism (Maleki and Eiteman, 2017). In *L. lactis*, in order to achieve efficient pyruvate accumulation, it is necessary to inactivate the pyruvate consuming pathways. In this study, we used the previously constructed strain CS4363 as a starting point, where genes encoding three lactate dehydrogenase (LDH) homologs (*ldh, ldhB, ldhX*), phosphotransacetylase (PTA) and alcohol dehydrogenase (ADHE) had been deleted (Solem et al., 2013; Liu et al., 2016a). After deleting these pathways, a strain mainly producing acetoin under aerobic conditions was obtained. To achieve pyruvate production, we further needed to inactivate the *als* gene, encoding α-acetolactate synthase (Figure 1). We attempted to delete the *als* gene using the genetic tool pCS1966, as described in the section Materials and Methods, which was challenging. However, after screening more than 800 colonies, we obtained the pyruvate-producing strain FS1072 (MG1363 Δ^3^*ldh* Δ*pta* Δ*adhE* Δ*als*) (Table 1).

**Figure 2:**
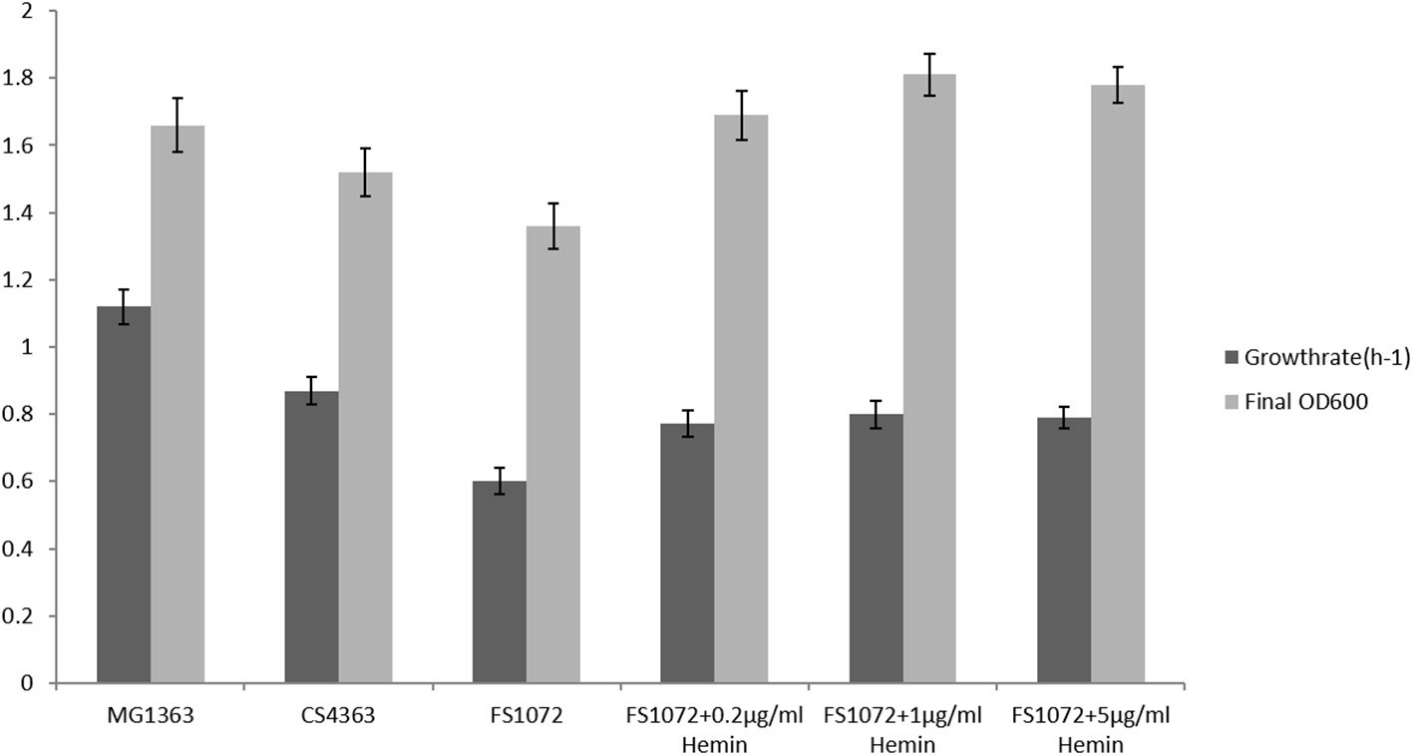
Growth characterization of the recombinant pyruvate-producing strain in rich medium. Strains were grown in M17 with 0.2% glucose. Measurements are the average value ± standard error from independent duplicate cultivations.

We tested the growth performance of the pyruvate-producing strain in rich medium M17 and minimal medium SAL. FS1072 relies on oxygen in order to grow, as oxygen is needed by the cytoplasmic NADH oxidase (NoxE) for regenerating NAD^+^, and we therefore examined the growth capacity of the engineered strain under aerobic conditions. As shown in Figure 2, both the specific growth rate and the final biomass yield of the recombinant strain FS1072 were lower than for the wild-type strain MG1363 and the strain CS4363, its immediate precursor. This indicated that the absence of a functional α-acetolactate synthase could have an adverse effect on growth. Normally, the H_2_O-forming NADH oxidase, NoxE, is responsible for the majority of NADH oxidase activity under aerobic conditions (Jensen et al., 2001). However, *L. lactis* also is capable of respiring, when hemin is added, which is a more efficient way to regenerate NAD^+^, improve biomass formation and help alleviate oxidative stress (Blank et al., 2001; Duwat et al., 2001; Koebmann et al., 2008). Indeed, the presence of hemin resulted in a significant increase of the growth capacity of the recombinant strain. As shown in Figure 2, the introduction of 0.2 μg/mL hemin significantly increased the growth rate of the recombinant strain FS1072. Higher hemin concentrations did not further improve growth rate or biomass formation. We also found that a high concentration of glucose inhibited the growth of strain FS1072, with 5% glucose having a slightly negative effect and 10% glucose significantly repressed the growth of the strain. This phenomenon was probably due to osmotic effects, why limiting the glucose content to 5% appeared to be a good choice.

**Figure 3:**
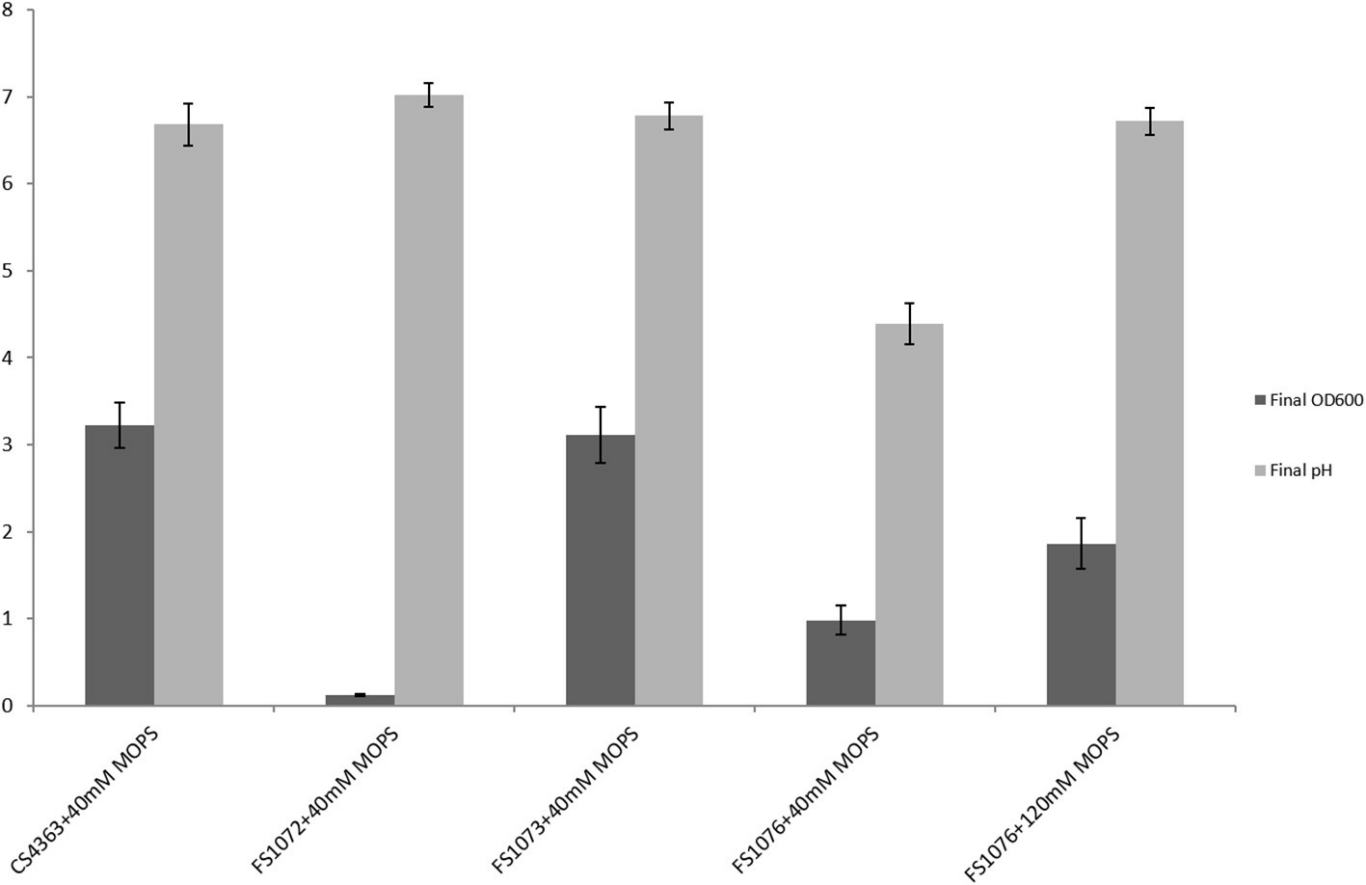
Growth characterization of the recombinant pyruvate-producing strain in minimal medium. Strains were grown in SAL with 1% glucose. Measurements are the average value ± standard error from independent duplicate cultivations.

In minimal medium SAL, as shown in Figure 3, the strain FS1072 could hardly grow, and the final cell density (OD_600_) was only 0.11. When we knocked-out the *als* gene, we had to use the defined medium SAL, as is deficient in pyrimidine compounds that interfere with the 5FO counterselection, and the poor growth of the *als* mutant on SAL medium probably explains why it was so difficult to obtain the mutant in the first place. To ascertain that deletion of the als gene caused the poor growth on SAL, we complemented FS1072 with a plasmid expressing *als*, pLC17, and obtained the strain FS1073 (MG1363 Δ^3^*ldh* Δ*pta* Δ*adhE* Δ*als* pLC17). As shown in Figure 3, the strain FS1073 had a drastically recovered growth capacity. Apparently, the absence of a functional α-acetolactate synthase had a significant effect on the cell growth in SAL medium. In an attempt to overcome the slow growth, we carried out a 21 days adaptive evolution experiment, and obtained the mutant strain FS1076 which grew better. Strain FS1076 was able to reach a cell density of 0.98 (OD_600_) in SAL containing 1% glucose and 40 mM MOPS buffer, and as high as 1.86 when 120 mM MOPS buffer was added, to compensate for the pH drop caused by pyruvate (Figure 3). In M17 medium, the growth capacity of strains FS1072 and FS1076 were quite similar.

### Transcriptomics Analysis With the Recombinant Pyruvate-Producing Strains

**Figure 4:**
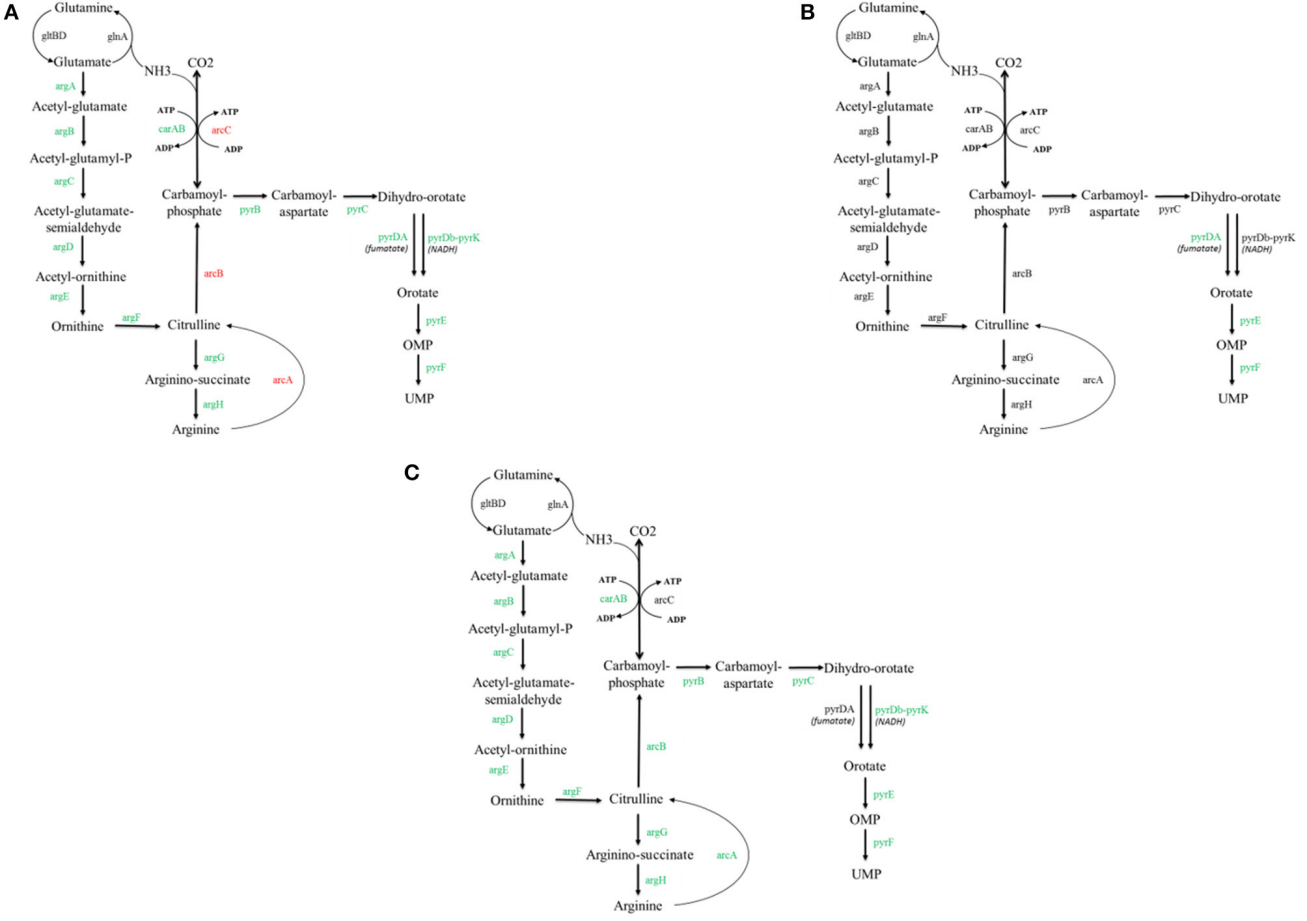
Transcriptomics analysis with the recombinant pyruvate-producing strain. Comparison of transcriptional level of selected genes for the FS1072 and FS1076 strains. All the genes involved in arginine metabolism and pyrimidine metabolism cycle are included. Red color represents up-regulated and Green color represents down-regulated in the metabolic network. **(A)** FS1072 in M17/SAL (SAL as control), **(B)** FS1076 in M17/SAL (SAL as control), **(C)** FS1076/FS1072 in SAL (FS1072 as control).

To find the reason for the improved growth of the mutant achieved after the adaptive evolution experiment, we compared the transcriptomes of FS1072 and FS1076 grown on the M17 and SAL medium, and for this purpose we relied on RNA-sequencing. A number of genes relevant to Arginine metabolism and pyrimidine metabolism were found to be differentially regulated under the various conditions (All the significantly expressed genes on the various conditions can be found in Supplementary Tables 1–4). The expression of 15 genes involved in these two pathways were downregulated in FS1072 in M17 medium as compared to in SAL medium (Figure 4A), which indicates that there are insufficient amounts of arginine in SAL medium. SAL medium does not contain pyrimidines. This conclusion was supported by the poor growth of FS1072 observed in SAL medium (Figure 3). However, the adapted strain FS1076, did not display any big differences between the two media (Figure 4B), which is reflected in the similar growth of FS1076 in these two media. In addition, we also compared the expression levels of genes between FS1072 and FS1076 in SAL medium (Figure 4C). The expression of almost all the genes involved in these two pathways in FS1076 down regulated compared to in FS1072, suggesting arginine and pyrimidine starvation in FS1072 in SAL medium. This observation correlated with the ability of FS1076 to grow better than FS1072 in SAL medium.

**Figure 5:**
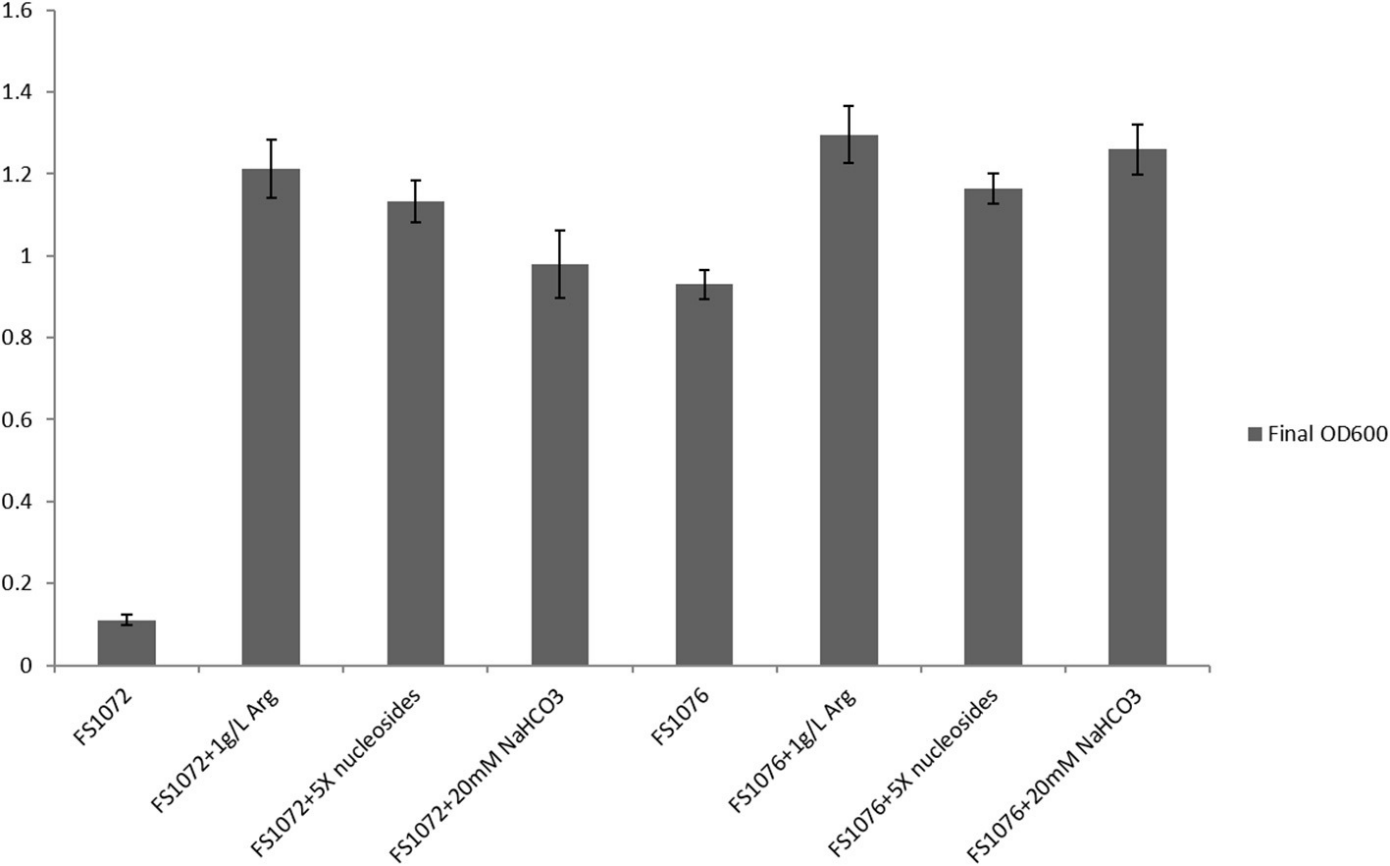
Growth characterization of the recombinant pyruvate-producing strain in minimal medium with different compounds. Strains were grown in SAL with 1% glucose. Measurements are the average value ± standard error from independent duplicate cultivations.

To experimentally verify the arginine and pyrimidine starvation indicated by the transcriptomic analysis, we supplemented the SAL medium with arginine and nucleosides, and recorded the effect of this on FS1072 and FS1076, respectively (Figure 5). As expected, by adding the two compounds, it was possible to increased the final cell density (OD_600_) of the two strains, in particular for FS1072, and furthermore, the specific growth rate of FS1072 approached that of FS1076. These results clearly demonstrated that FS1072 was starved for arginine and pyrimidines in SAL medium, however, the underlying reason remained unclear. After inspecting the metabolic pathways for arginine and pyrimidine, we speculated that the poor growth of FS1072 might be due to insufficient CO_2_. CO_2_ is necessary for cell growth, and when *als* is knocked out in the CS4363, this would block a source of CO_2_ production (Figure 1). To test this hypothesis, we added 20 mM NaHCO_3_ into the SAL medium, and as seen in Figure 5, this had a beneficial effect on the growth of FS1072 and FS1076, similar to the effect of arginine and nucleosides (Figure 5). We thus conclude that knocking out *als* leads to deprivation of CO_2_, and that this is the reason for why it was so difficult to isolate *als* mutants on SAL medium.

### Pyruvate Production From Glucose in a Bioreactor With pH Control

**Figure 6:**
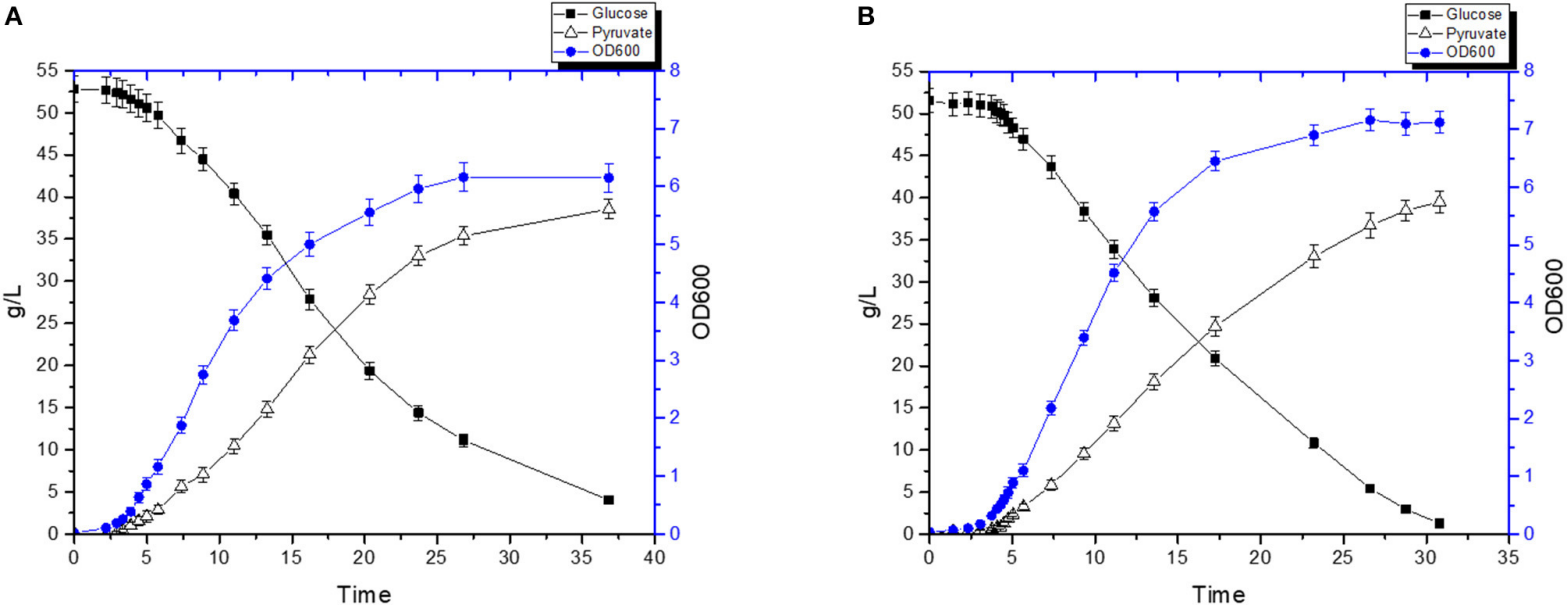
Pyruvate production by the recombinant strain FS1076. The recombinant strain FS1076 was grown in M17 with 5% glucose. **(A)** 0 μg/mL hemin, **(B)** 1 μg/mL hemin. Measurements are the average value ± standard error from independent duplicate cultivations.

We tested the efficiency of our new pyruvate platform under controlled settings using a bioreactor (Figure 6). Without hemin, FS1076 produced 38.6 g/L pyruvate in 36.8 h (Figure 6A) from an initial glucose concentration of 52.8 g/L and around 4 g/L glucose remained in the broth. The pyruvate productivity was 1.05 g/L·h and the yield was 80.8% of the theoretical maximum. In contrast, FS1076 with hemin had higher pyruvate productivity. As Figure 6B shows, FS1076 with 1 μg/mL hemin resulted in 39.51 g/L pyruvate in 30.8 h, corresponding to a productivity of 1.28 g/L·h and a yield of 80.4%. Thus, hemin was clearly beneficial for reducing the fermentation time and thereby increasing productivity. These results demonstrate that the yield of pyruvate for FS1076 was high and despite a very low inoculum (OD_600_ only 0.038), we achieved a good pyruvate productivity. Pyruvate production in different strains, especially *E. coli*, has been extensively investigated (Zhu et al., 2008; Maleki and Eiteman, 2017; Yang and Xing, 2017). Zhu et al. reported the highest pyruvate concentration achieved using *E. coli* in defined medium, 90 g/L pyruvate with an overall productivity of 2.1 g/L·h and yield of 0.68 g/g (Zhu et al., 2008). Compared to the *E. coli* strain, the titer we achieved in this study is not the highest, but our yield is higher. Looking into the setup used by Zhu et al. reveals that they relied on fed-batch fermentation with a high initial cell density of 3 (OD600), where our initial cell density was only 0.038 (OD600). In addition the high titer Zhu et al. obtained required feeding of a mixture of acetate & glucose, which is far more complicated than the simple setup used here. In order to establish cost-efficient biotechnological production of pyruvate, it is not only necessary to have a fermentation process that delivers a high yield, high titer, and a high productivity. It is an obvious advantage if the fermentation process is simple, as it is in our case.

**Figure 7:**
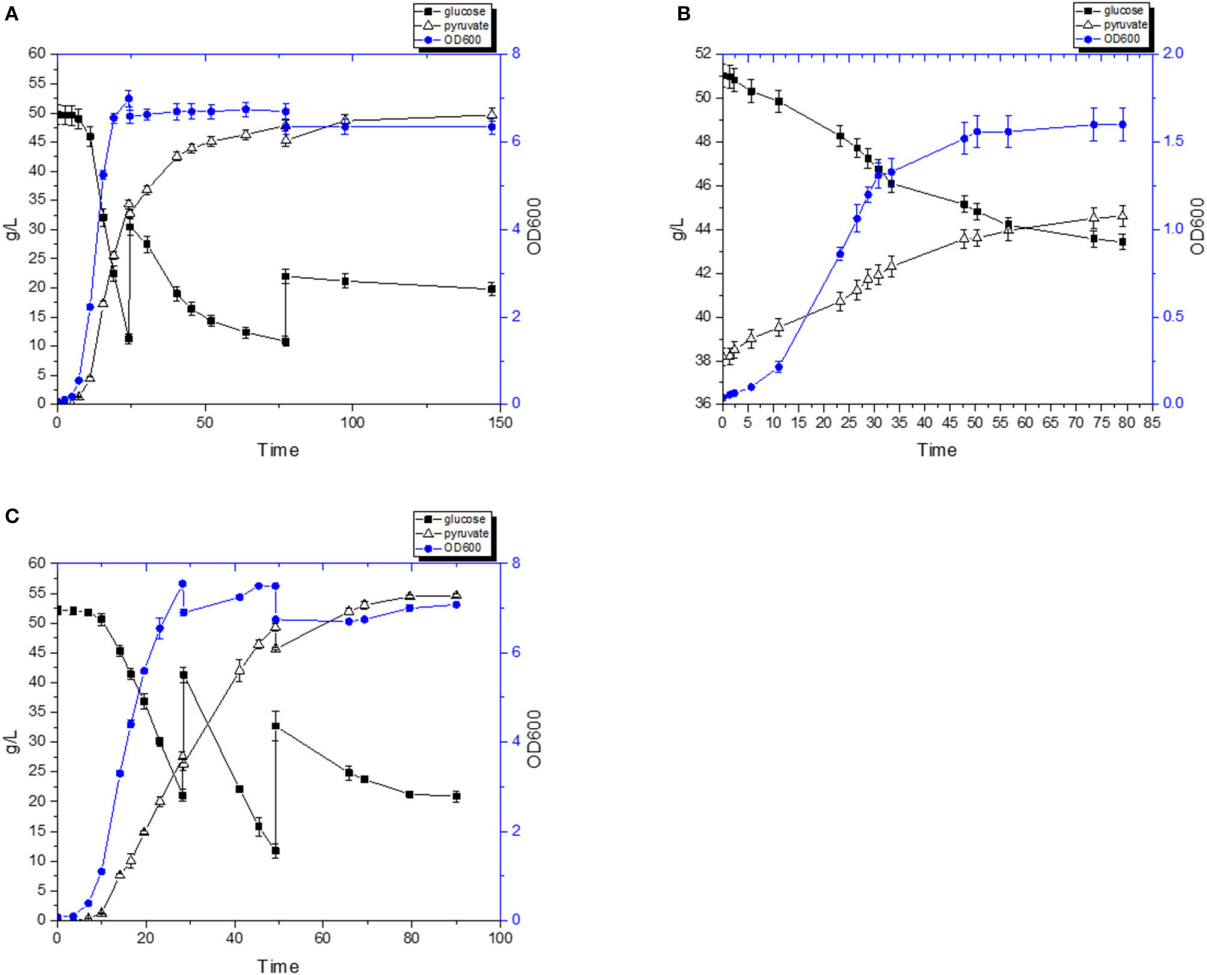
Pyruvate production by the recombinant strain FS1076. The recombinant strain FS1076 was grown in M17 medium. **(A)** Fed-batch culture with 1 μg/mL hemin. **(B)** Fermentation with an initial 38 g/L of pyruvate. **(C)** Fed-batch culture with 1 μg/mL hemin with 10 mM betaine. Measurements are the average value ± standard error from independent duplicate cultivations.

In an attempt to further increase the pyruvate titer, a fed-batch fermentation strategy was devised (Figure 7A). After 50 h of fermentation, pyruvate production had almost ceased, and after 150 h the titer was 49.7 g/L. We speculated that the main limiting factor for achieving higher pyruvate titer probably was a high osmotic pressure or product inhibition. We tested this by carrying out a fermentation experiment where 38 g/L of pyruvate was present from the beginning. As shown in Figure 7B, both the specific growth rate and final biomass of the strain FS1076 were significantly repressed by the pyruvate present in the medium. The specific growth rate and final biomass only reached 0.13 h^−1^ and 1.6, respectively. Moreover, the final concentration of pyruvate, including the initial pyruvate, was only 46 g/L. Therefore, we speculate that the tolerance of pyruvate or high osmotic stress maybe the limiting factor preventing further accumulation of pyruvate. An explanation for the observed termination of cell growth and pyruvate formation is the high osmotic stress caused by high concentration of pyruvate as well as the Na^+^ accumulated due to NaOH added to maintain the pH. To overcome this problem, many microorganisms can accumulate organic osmolytes, such as betaine, which naturally protect them from high osmotic stress (Yancey, 2005). By adding betaine to the fermentation medium, various fermentation processes have been optimized, e.g., production of pyruvate (Zhu et al., 2008), ethanol (Underwood et al., 2004), and lactate (Zhou et al., 2006). Thus, we tested whether the compatible solute betaine could have a beneficial effect in a fed-batch fermentation setup. After optimization, as shown in Figure 7C, with the help of 10 mM betaine, the growth of the pyruvate strain was improved and after 90 h the pyruvate titer reached 54.6 g/L which is 10% higher than without betaine. This is encouraging but the reasons for the benefits of betaine in this case are still unclear. We believe that adaptive evolution is a good approach for obtaining mutants with improved tolerance to osmotic stress, and that pyruvate production can be further improved by using these mutants.

### Pyruvate Production From Lactose Contained in Dairy Waste

Recent trends toward the production of green chemicals calls for processes that are efficient, cost-effective and sustainable, and an important element is to find cheap and renewable feedstocks. Residual whey permeate (RWP), which is a lactose rich side stream generated by the dairy industry, is a good candidate as a cheap carbon source for producing food-grade pyruvate. Strain FS1076 is a derivative of the plasmid-free laboratory strain MG1363, which lacks the ability to metabolize lactose, and to restore this ability, we therefore introduced the lactococcal plasmid pLP712 into strain FS1076 to generate FS1080.

**Figure 8:**
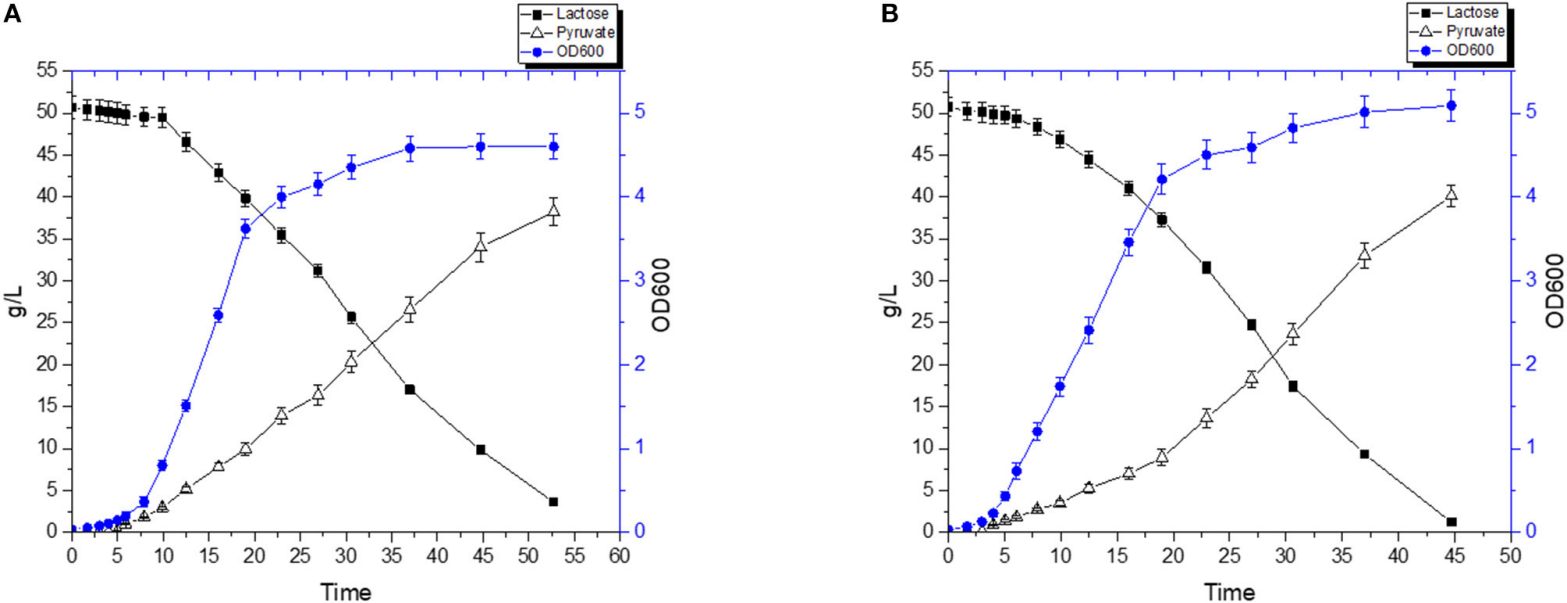
Pyruvate production with RWP and 2% Yeast extract. The recombinant strain FS1080 was grown in the diluted whey with 2% yeast extract. **(A)** 0 μg/mL hemin, **(B)** 1 μg/mL hemin. Measurements are the average value ± standard error from independent duplicate cultivations.

A fermentation was carried out using a combination of diluted RWP and 2% (w/v) yeast extract. FS1080 produced pyruvate as main product during the fermentation. Figure 8 shows that, without hemin, FS1080 produced 38.21 g/L pyruvate in 52.7 h and with a yield of 78.9% (Figure 8A). Adding 1 μg/mL hemin resulted in 40.1 g/L pyruvate in 44.7 h with a yield of 78.6% (Figure 8B). Thus, the pyruvate productivity using RWP with 2% yeast extract added was slightly lower compared to what was achieved in M17 medium containing glucose.

**Figure 9:**
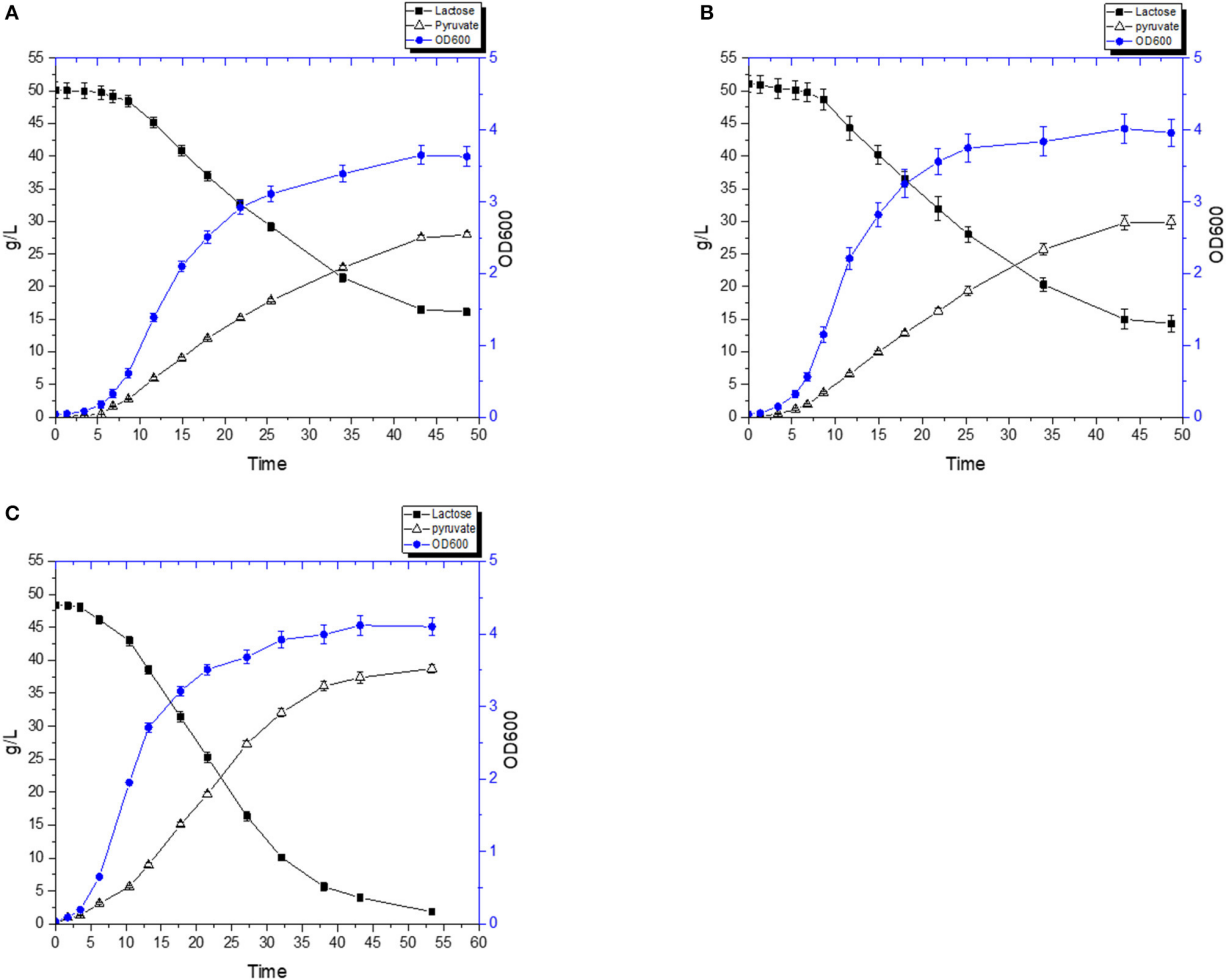
Pyruvate production with 0.5% Yeast extract. The recombinant strain FS1080 was grown in the diluted whey with 0.5% yeast extract. **(A)** 0 μg/mL hemin, **(B)** 1 μg/mL hemin, **(C)** 1 μg/mL hemin with 2 μg/L CoCl_2_.6H_2_O with 2 μg/L MnSO_4_.H_2_O. Measurements are the average value ± standard error from independent duplicate cultivations.

To further reduce the cost of the medium, we also carried out the fermentation using 0.5% (w/v) yeast extract. Without hemin, the pyruvate titer reached 27.9 g/L in 48.6 h with a yield of 79.9% (Figure 9A), whereas FS1080 with 1 μg/mL hemin resulted in a pyruvate titer of 29.9 g/L in 48.6 h with a yield of 79.1% (Figure 9B). Compared with the fermentation where 2% YE was used, the pyruvate yield obtained using 0.5% YE was very similar, but since FS1080, when grown with 0.5%YE, did not consume all the sugar in the medium, the final titer of pyruvate was much lower. To solve this problem, we tried to add various components to the fermentation medium, including different metal ions. Shorb reported growth stimulation of *L. lactis* by adding crystalline vitamin B12 (Shorb, 1948). Cobalt is at the center of the vitamin B12 complex, and is an essential factor for growth of a number of lactic acid bacteria (Hoff-Jorgensen, 1949). Manganese has also been found to be essential for many lactic acid bacteria, and manganese plays an important role in many enzymes, including oxidoreductases, transferases, hydrolases, lyases, ligases, and isomerases (Archibald, 1986). Eventually we found that adding a low concentration of the two metal ions Co^2+^ and Mn^2+^ improved the growth and sugar consumption. Thus, by including 1 μg/mL hemin, 2 μg/L CoCl_2_∙6H_2_O and 2 μg/L MnSO_4_∙H_2_O, the lactose in medium could almost completely be consumed, and the pyruvate titer was enhanced to 38.7 g/L in 53.4 h with a yield of 80.9% (theoretical maximum) (Figure 9C), similar to the outcomes under glucose. The high cost of yeast extract (4000 $/ton, Angel Yeast China), could be a limiting factor for the process described here, e.g., when using 0.5% YE the cost of the fermentation medium alone would amount to $0.5 per kg pyruvate produced (assuming that RWP is zero cost). It is likely that by re-using the cells, the fermentation medium associated costs could be further reduced, which we will explore in the future.

## Conclusion

In this study, *L. lactis* was engineered as a new cell factory yielding pyruvate and a fermentation based on a cheap renewable feedstock was developed. The engineered *L. lactis* can convert different sugars into pyruvate efficiently. The results demonstrate that *L. lactis* has a good potential as a cell factory for producing pyruvate. It is possible to achieve sustainable and cost-efficient bioconversion of waste products from the dairy industry (RWP) to pyruvate, and the process developed shows great potential for commercial realization.

## Data Availability Statement

The datasets presented in this study can be found in online repositories. The names of the repository/repositories and accession number(s) can be found at: https://www.ncbi.nlm.nih.gov/bioproject/PRJNA669338/.

## Author Contributions

PJ, CS, JL, JC, and FS designed the experiments and revised the manuscript. FS performed this experiment and wrote this manuscript. XL worked on RNAseq analysis. All authors read and approved the final manuscript.

## Conflict of Interest

The authors declare that the research was conducted in the absence of any commercial or financial relationships that could be construed as a potential conflict of interest.
