# Supplementary material for: Efficient Production of Pyruvate Using Metabolically Engineered Lactococcus lactis
Source: Front Bioeng Biotechnol. 2021 Jan 6;8:611701. doi: 10.3389/fbioe.2020.611701 (PMC7815928; doi:10.3389/fbioe.2020.611701)
Supplement: Supplementary file 2 [file Table_5.docx]

**Table 5 Composition of M17 and SAL media**

| **﻿Constituent** | **﻿Concentration in medium M17** |
| --- | --- |
| ascorbic acid  lactose  magnesium sulfate  meat extract  meat peptone(peptic)  sodium glycerophosphate  soya peptone(papainic)  tryptone  yeast extract | 0.5g/L  5g/L  0.25g/L  5g/L  2.5g/L  19g/L  5g/L  2.5g/L  2.5g/L |
| **Constituent** | **Concentration in medium SA** |
| L-Alanine | 3.4 mM |
| L-Arginine  L-Asparagine  L-Cysteine  L-Glutamate  L-Glutamine  Glycine  L-Histidine  L-Isoleucine  L-Leucine  L-Lysine-HCl  L-Methionine  L-Phenylalanine  L-Proline  L-Serine  L-Threonine  L-Tryptophan  L-Tyrosine  L-Valine  NH4Cl  K2SO4  KH2PO4  Na-acetate  MOPS  Tricine  CaCl2  MgCI2  FeSO4  NaCl  Vitamins^a^ | 1.1 mM  0.8 mM  0.8 mM  2.1 mM  0.7 mM  2.7 mM  0.3 mM  0.8 mM  0.8 mM  1.4 mM  0.7 mM  1.2 mM  2.6 mM  2.9 mM  1.7 mM  0.5 mM  0.3 mM  0.9 mM  9.5 mM  0.28 mM  1.3 mM  15 mM  40 mM  4 mM  0.0005 mM  0.52 mM  0.01 mM  50 mM  + |
| Micronutrients^b^ | + |

﻿^a^Vitamins: 0.4 μM biotin, 10 μM pyridoxal-HCl, 2.3 μM folic acid, 2.6 μM riboflavin, 8 μM niacinamide, 3 μM thiamine-HCl, and 2 μM pantothenate.

^b^Micronutrients: 0.003 μM (NH_4_)_6_(MO_7_)_24_, 0.4 μM H_3_BO_3_, 0.03 μM CoCl_2_, 0.01 μM CuSO_4_, 0.08 μM MnCl_2_, and 0.01 μM ZnSO_4_.

SAL medium: SA medium with Na-Acetate replaced by 2mg/L Alpha-lipoic acid
